# Probing excitations and cooperatively rearranging regions in deeply supercooled liquids

**DOI:** 10.1038/s41467-023-37793-2

**Published:** 2023-05-05

**Authors:** Levke Ortlieb, Trond S. Ingebrigtsen, James E. Hallett, Francesco Turci, C. Patrick Royall

**Affiliations:** 1 H.H. Wills Physics Laboratory, Tyndall Avenue, Bristol, BS8 1TL UK; 2 Centre for Nanoscience and Quantum Information, Tyndall Avenue, Bristol, BS8 1FD UK; 3 DNRF Centre for Glass and Time, IMFUFA, Department of Science, Systems and Models, Roskilde University, Postbox 260 DK–4000, Roskilde, Denmark; 4 Department of Chemistry, School of Chemistry, Food and Pharmacy, University of Reading, Whiteknights, PO Box 224 Reading RG6 6AD, UK; 5 School of Chemistry, University of Bristol, Cantock’s Close, Bristol, BS8 1TS UK; 6 Gulliver UMR CNRS 7083, ESPCI Paris, Université PSL, 75005 Paris, France

**Keywords:** Statistical physics, Statistical mechanics, Phase transitions and critical phenomena, Structure of solids and liquids

## Abstract

Upon approaching the glass transition, the relaxation of supercooled liquids is controlled by activated processes, which become dominant at temperatures below the so-called dynamical crossover predicted by Mode Coupling theory (MCT). Two of the main frameworks rationalising this behaviour are dynamic facilitation theory (DF) and the thermodynamic scenario which give equally good descriptions of the available data. Only particle-resolved data from liquids supercooled below the MCT crossover can reveal the microscopic mechanism of relaxation. By employing state-of-the-art GPU simulations and nano-particle resolved colloidal experiments, we identify the elementary units of relaxation in deeply supercooled liquids. Focusing on the excitations of DF and cooperatively rearranging regions (CRRs) implied by the thermodynamic scenario, we find that several predictions of both hold well below the MCT crossover: for the elementary excitations, their density follows a Boltzmann law, and their timescales converge at low temperatures. For CRRs, the decrease in bulk configurational entropy is accompanied by the increase of their fractal dimension. While the timescale of excitations remains microscopic, that of CRRs tracks a timescale associated with dynamic heterogeneity, ${t}^{*} \sim {\tau }_{\alpha }^{0.8}$. This timescale separation of excitations and CRRs opens the possibility of accumulation of excitations giving rise to cooperative behaviour leading to CRRs.

## Abstract (web summary)

Experimental data of the transition of a supercooled liquid into glass is compatible with both dynamic and thermodynamic theories. Here the authors use experiments and MD simulations at very low temperatures to show that both theories are connected.

## Introduction

Cool or compress any liquid fast enough, and it will not order into a crystal but remain a liquid before it eventually falls out of equilibrium and becomes a *glass*. Over the years, a variety of theoretical approaches have been developed to account for the slowdown in the dynamics of such supercooled liquids as they approach the experimental glass transition temperature *T*_*g*_^1–3^. What happens below *T*_*g*_ remains a matter of conjecture, as equilibration is not possible, with wildly differing theoretical standpoints which nevertheless provide equally good interpretations of the available data^1,4^. These can be broadly classified into those which presume that the dramatic slowdown in supercooled liquids is predominantly a *dynamic* phenomenon and those which invoke a thermodynamic origin^1–3^.

Among the former is dynamic facilitation^5,6^, in which the arrest is attributed to the emergence of kinetic constraints in the liquid and focuses on dynamic heterogeneities^1^. Dynamic facilitation is built around the idea of a dynamical phase transition^6^. Unlike conventional thermodynamic phase transitions, where the coexisting phases are characterised by distinct static properties, the dynamical transition amounts to the coexistence of mobile regions where the particles relax on a microscopic timescale and immobile regions where relaxation is very slow. The mobile regions feature *excitations* which constitute the elementary units of relaxation in dynamic facilitation.

By contrast, a number of theoretical approaches have been developed which emphasise the role of thermodynamic properties, expressed through the micro-structure assumed by the constituent particles. While the two-point structure, (i.e. the pair correlation function) changes little, higher-order correlations exhibit a marked change upon approaching the glass transition^7^. In particular, Adam–Gibbs theory and random first-order transition (RFOT) theory emphasise configurational entropy—which drops progressively as the temperature is reduced—and the role of so-called cooperatively rearranging regions (CRRs). These constitute groups of mobile particles through which the system relaxes. These CRRs exhibit only a few distinct states. For a fixed number of states per CRR, the drop in configurational entropy means that CRRs are expected to grow in size as the system approaches the glass transition. In this way, the dynamics of the mobile particles in the CRRs are intimately coupled to a thermodynamic quantity, configurational entropy. Other theories, such as geometric frustration, emphasise the correlation between growing numbers of locally favoured structures and the emergence of slow dynamics^8^, which have been identified with a drop in configurational entropy^9,10^.

Accessing equilibrated low-temperature (or high density for repulsive systems) static and dynamic data is key to discriminating between competing theories, as their features differ more markedly under these conditions. For some time, computational and experimental techniques able to discriminate between theoretical predictions using many-body spatial and temporal correlations of particle-resolved quantities were limited to the *T* ≳ *T*_mct_ regime of weak supercooling for computer simulations, and volume fraction *ϕ* ≲ *ϕ*_mct_ for particle-resolved studies of colloidal systems^11,12^, where MCT stands for the crossover of mode-coupling theory. Since relaxation for *T* ≳ *T*_mct_ is well described by MCT, accessing *T* ≲ *T*_mct_ is crucial to probe which postulated relaxation mechanism is dominant. For example, RFOT predicts a quantitative change in the mechanism of relaxation at the mode-coupling crossover, which may be interpreted as the high-temperature limit of stability of the glassy state^13^ related to the disappearance of the glassy minimum of the Franz-Parisi potential of replica theory^14,15^.

Recently, it has become possible to access equilibrated configurations for *T* ≲ *T*_mct_ with the advent of SWAP Monte Carlo computer simulations^16^. With this approach, theories related to static quantities (such as RFOT) have been tested with some success^16–19^, while dynamic quantities have largely been investigated in the higher temperature regime *T* ≳ *T*_mct_^20^. Again, some success of the predictive power of dynamic facilitation was found^20,21^. Quantitative analysis contrasting the key objects of RFOT (e.g. the cooperatively rearranging regions) and dynamic facilitation (i.e. the elementary excitations) in the relevant regime of deep supercooling is lacking to date, and this is the focus of the present work.

We exploit two new techniques to obtain dynamic particle-resolved data in the regime beyond the mode-coupling crossover *T* < *T*_mct_ to probe the predictions of thermodynamic and dynamic theories. The first of these developments is highly-efficient GPU simulations using the Roskilde University Molecular Dynamics (RUMD) package^22^ on dedicated graphical processing units. The simulations provide a complete static and dynamical picture that forms much of our analysis. The second advance is nanoparticle-resolved studies of colloidal systems^10^, which by using smaller colloids takes advantage of the Brownian timescale of colloidal systems *τ*_*B*_ ~ *σ*^3^, where *σ* is the particle diameter to speedup diffusion and access longer timescales to obtain colloidal hard-sphere data at *ϕ* ≳ *ϕ*_mct_. Both techniques deliver dynamic data for state points where the structural relaxation time is around a thousand times that at the mode-coupling crossover^10,23^.

These techniques thus provide deeply supercooled data, which is suitable to test the different scenarios for dynamic facilitation and RFOT, respectively. We focus on three key questions: (i) does the nature of excitations remain unchanged at deep supercooling, as predicted by dynamic facilitation? (ii) do the CRRs become significantly more compact? (iii) Finally, is there a connection between excitations and CRRs that depends on the degree of supercooling? We provide a detailed numerical study of a binary Lennard-Jones Kob-Andersen mixture and further insight from nanoparticle-resolved experiments using hard-sphere–like colloids^9,10^. The simulations allow us to study both the microscopic timescales of excitations and longer timescales associated with cooperative rearrangements. The colloidal data provide an experimental comparison of the spatial features of the cooperative rearrangements. The simulations are performed at a large-to-small particle composition of 2:1 and 3:1 for their enhanced stability against crystallisation compared to the often-used 4:1 composition^23^. Unless otherwise specified, we present data from the 2:1 composition and *N* = 10,002 system size. Throughout, we use Lennard-Jones units for the larger particles for simulation data. For the experiments, we express time in Brownian times *τ*_*B*_ and length in diameter *σ*. Experiment and computer simulation details are in the materials and methods and supplementary information (SI).

In its simplest conception, our approach is to seek to determine whether liquids supercooled past the mode-coupling crossover relax via excitations or through cooperatively rearranging regions. To do so, we base our analysis on methods developed previously, in particular, that of ref. ^20^ to identify excitations. In the case of CRRs, we follow two analyses. Firstly, one can choose some proportion of particles which have moved the furthest on an appropriate timescale and interpret these as being in a CRR^24^. Secondly, early work considering dynamic heterogeneity found string-like motion in the *T* > *T*_mct_ regime^25^. Here we use this “string analysis” as a second method to probe CRRs. Finally, we consider geometric motifs (locally favoured structures) associated with slow dynamics and their relationship with excitations and CRRs.

For these analyses, we require particle-resolved data sampled at differing rates. The excitations are identified on microscopic timescales, and we run short simulations for this purpose. For the CRRs, the timescales are much longer. In the case of the experimental data that we present, we have optimised the data acquisition for CRRs, and thus the limited number of frames accessible (around 100 per dataset) means that these are sampled at a timescale rather longer than that associated with excitations, and so, therefore, we focus the analysis of our experimental data on the CRRs. The string analysis also requires a higher frame rate, so here too, we focus on the simulation data. We furthermore compute the configurational entropy for the simulations data via thermodynamic integration^26^.

## Results

Our results are organised as follows: Firstly, we present the general relaxation behaviour of the systems considered, reporting structural relaxation times and measures of dynamical heterogeneity. Secondly, we determine the population of excitations in the supercooled liquids and test the predictions of dynamic facilitation; Thirdly, we further characterise the simulated liquids with their bulk configurational entropy, testing the Adam–Gibbs scenario, which employs the concept of cooperatively rearranging regions (CRR). Then, we operatively define such regions in two ways (i) by using the displacement distribution and (ii) by identifying large clusters of string-like motion. We then connect the excitation and the CRR analyses and quantitatively compare the spatial distributions of CRRs and excitations by characterising their cross-correlations. Finally, we compare CRRs and excitations with locally favoured structures related to the dynamical arrest.

### Dynamical particle-resolved data at deep supercooling

**Fig. 1:**
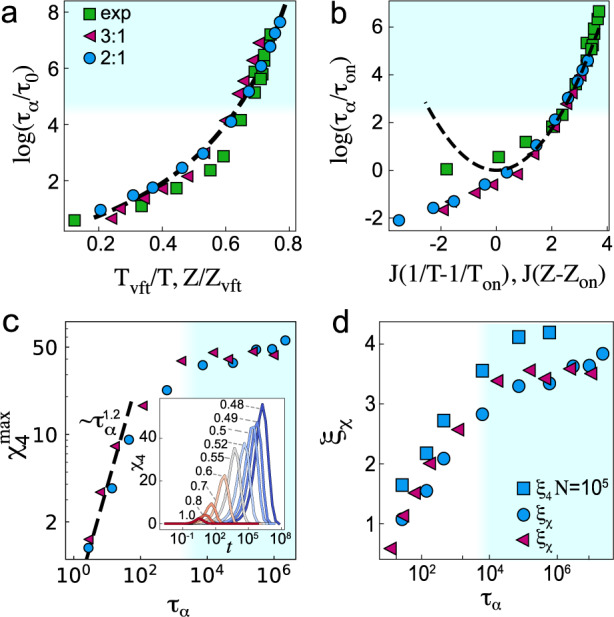
Relaxation well below the mode-coupling crossover. Blue shading indicates state points past the mode-coupling crossover. **a** Angell plot of relaxation time *τ*_*α*_ plotted against temperature *T* and compressibility factor *Z*. scaled with Vogel–Fulcher–Tamman (VFT) parameters (Eq. (1)). The dashed line is the VFT fit for Kob–Anderson (KA) 2:1. The KA data are plotted with respect to *T*_vft_, as is the fit while the experimental data are plotted with respect to *Z*_vft_. The legend in (**a**) also applies to (**b**–**d**), although no experimental data are shown in (**c**, **d**). **b** Relaxation time scaled with parameters of the parabolic fit (Eq. (2)). The dashed line is the parabolic fit for KA 2:1. Similarly to (**a**), the KA data are plotted with respect to *T*_vft_, as is the fit while the experimental data are plotted with respect to *Z*_vft_. Blue shading indicates state points past the mode-coupling crossover. **c** Maximum of dynamic susceptibility *χ*_4_ vs. *τ*_*α*_, compared with the ${\tau }_{\alpha }^{1.2}$ scaling seen previously^16, 31^ (black dashed line). Inset: dynamic susceptibility *χ*_4_(*t*) for different temperatures for the KA 2:1 system. **d** Dynamic lengthscale inferred from the maximum of dynamic susceptibility ${\chi }_{4},{\xi }_{\chi }={({\chi }_{4}^{\max })}^{3.0}$^32^ and four-point dynamic lengthscale *ξ*_4_ evaluated for larger system size. Blue shading indicates state points past the mode-coupling crossover. Specifically, these values are *T*_mct_ = 0.55 (0.589) and 0.70 (0.640) for KA 2:1 and 3:1, respectively, and *Z*_mct_ = 25.1 (0.689) for the experiments. Numbers in brackets denote the scaling by the VFT point as used in (**a**).

In Fig. 1a, we plot the structural relaxation time *τ*_*α*_ against the control parameter, which is the inverse temperature 1/*T* and the compressibility factor *Z*^9,27^ for simulations and experiments, respectively. We determine *τ*_*α*_ as described in the SI, section IIA. and fit it with the Vogel–Fulcher–Tamman (VFT) equation,

1 $${\tau }_{\alpha }^{{{{{{{{\rm{sim}}}}}}}}}={\tau }_{0}\exp \left(\frac{D{T}_{{{{{{{{\rm{vft}}}}}}}}}}{T-{T}_{{{{{{{{\rm{vft}}}}}}}}}}\right)\quad {\tau }_{\alpha }^{\exp }={\tau }_{0}\exp \left(\frac{D{Z}_{{{{{{{{\rm{vft}}}}}}}}}}{{Z}_{{{{{{{{\rm{vft}}}}}}}}}-Z}\right)$$

for simulations and experiments, respectively. Here *τ*_0_ is a microscopic timescale, and *D* represents the fragility. We fit the data to obtain the temperature *T*_vft_ and reduced pressure *Z*_vft_ at which the relaxation time is predicted to diverge. Note that this expression has been related to some thermodynamic interpretations^28^. In contrast, Fig. 1b shows that the same data can be described equally well by the parabolic law of dynamic facilitation, which reads as

2 $${\tau }_{\alpha }^{{{{{{{{\rm{sim}}}}}}}}}={\tau }_{{{{{{{{\rm{on}}}}}}}}}\exp \left\{{J}^{2}{\left(\frac{1}{T}-\frac{1}{{T}_{{{{{{{{\rm{on}}}}}}}}}}\right)}^{2}\right\}\quad {\tau }_{\alpha }^{\exp }={\tau }_{{{{{{{{\rm{on}}}}}}}}}\exp \left\{{J}^{2}{\left(Z-{Z}_{{{{{{{{\rm{on}}}}}}}}}\right)}^{2}\right\}$$

for simulations and experiments, respectively. Here, *J*, *T*_on_ and *Z*_on_ are characteristic energy and the onset point of heterogeneous dynamics. As previously observed in refs. ^4,29^, there is no immediate reason to prefer one description to the other, although, for the deepest supercooling considered, the parabolic fit appears somewhat better for our data. We also estimate the mode-coupling crossover (MCT) empirically via a power-law fit of the relaxation times; the values are noted in the caption of Fig. 1. The shading across the figures illustrates that our most deeply supercooled data have relaxation times about 1000 times larger than systems at the MCT crossover. Details of the fits for the simulations are in the SI, Fig. S2 and in ref. ^9^ for the experiments.

To investigate the dynamic heterogeneity of our system, we consider the dynamic susceptibility *χ*_4_(*t*). Our method to determine *χ*_4_(*t*) is provided in the SI (section II), note that we use an ensemble-dependent *χ*_4_(*t*), which may influence the values that we obtain^30^. We plot the dynamic susceptibility in Fig. 1c inset, indicating that the dynamical heterogeneity increases with supercooling through the range of state points that we access. In Fig. 1c, we show the peak value of the dynamic susceptibility ${\chi }_{4}^{\max }$. This is a measure of the number of particles involved in dynamically heterogeneous regions. When we plot ${\chi }_{4}^{\max }$ as a function of the relaxation time, we see a behaviour consistent with a crossover at a temperature slightly higher than the mode-coupling temperature *T*_mct_ ≈ 0.55 and a slower growth at deeper supercooling. This is compatible with the RFOT prediction of a change in relaxation behaviour around *T*_mct_. For weaker supercooling *T* ≳ *T*_mct_, we see behaviour consistent with the scaling ${\chi }_{4}^{\max } \sim {\tau }_{\alpha }^{1.2}$ as obtained previously in ref. ^31^.

The peak of the dynamic susceptibility may be understood to represent the number of particles characteristic of dynamic heterogeneity and has been related to a dynamic lengthscale ${\xi }_{\chi }={({\chi }_{4}^{\max })}^{1/3.0}$, where the exponent 3.0 is that obtained by ref. ^32^. In Fig. 1d, we see that the dynamic lengthscale increases significantly in the temperature range sampled, although the rate of increase drops at deep supercooling.

### Identifying excitations

**Fig. 2:**
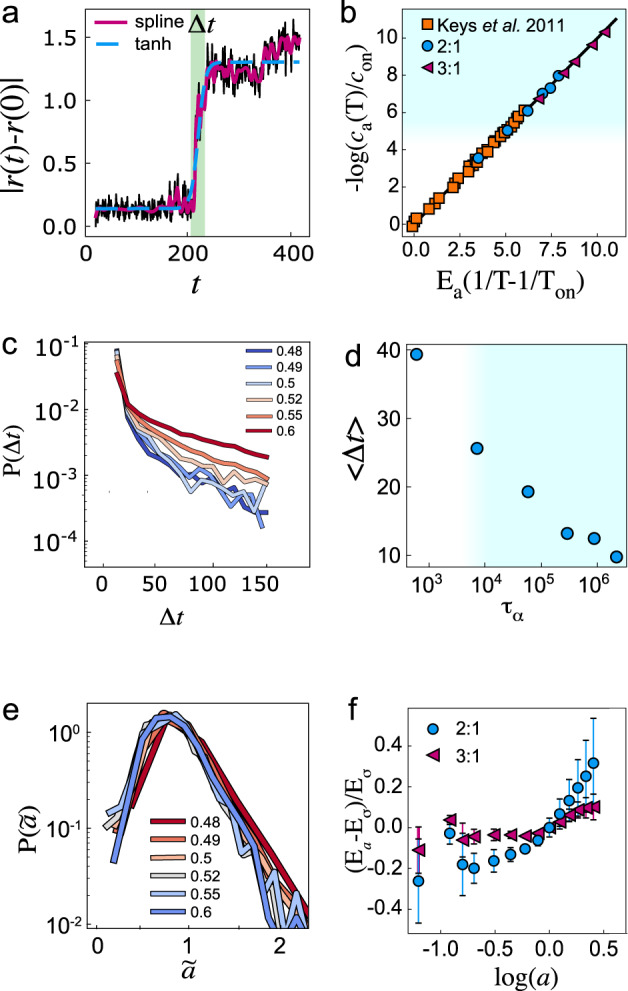
Testing the predictions of dynamic facilitation. Blue shading indicates state points past the mode-coupling crossover. **a** Single-particle displacement to illustrate the algorithm to identify excitations (see SI Section IIC for details). The green shading represents the width of the tanh fit from which the duration of the excitation is found. **b** Scaled temperature dependence of the concentration of excitations in comparison with the data by ref. ^20^. **c** Probability distribution of excitation durations Δ*t*. **d** Mean of the *P*(Δ*t*) distribution. **e** Distribution of displacements of particles identified in excitations. The magnitude of the displacements $\tilde{a}$ is determined from the fit to the displacements shown in (**a**). **f** Energy scaling as a function of excitation size. Here the parameter *γ* = 1, and *E*_*σ*_ = *E*_*a*_(*a* = *σ*). Error bars are standard deviations from fits such as indicated in Supplementary Fig. 5. Further details of the analysis may be found in ref. ^20^.

We focus first on the identification of excitations in the simulated liquids. To do so, we describe a procedure to identify particles in excitations, inspired by ref. ^20^. We set a timescale *t*_*a*_ = 200 and a lengthscale *a* = 0.5 and a trajectory of length *t*_traj_ = 5*t*_*a*_ generated from equilibrated configurations. To ensure that a given particle commits to the new position, the average position of an eligible particle in the first and the last *t*_*a*_ of the trajectory is required to be >*a*. To identify when the excitation occurs, we check in each frame if the average position of the particle during the previous and following *t*_*a*_ are at least *a* apart. The time at which the excitation takes place and its duration Δ*t* are determined using a hyperbolic tangent fit of the displacement after smoothing the trajectory with a spline [Fig. 2a] (see SI section IIC for further details). We remark that the jump dynamics is well-defined at low temperatures, in particular below the MCT crossover. Hence, our algorithm is well-suited to identify excitations in this regime, while at higher temperatures, there are relaxation events that do not satisfy the criteria above.

We express the population of excitations *c*_*a*_ as the fraction of particles identified as excitations during a trajectory of length *t*_traj_. We find the expected scaling of the population of particles in excitations:

3 $${c}_{a}={c}_{{{{{{{{\rm{on}}}}}}}}}\exp \left[-{E}_{a}\left(\frac{1}{T}-\frac{1}{{T}_{{{{{{{{\rm{on}}}}}}}}}}\right)\right]$$

in Fig. 2b which is consistent with ref. ^20^. Here, the energy scale for *a* = 0.5 related to the excitation population ${E}_{a}^{2:1}=10.5\pm 0.6$ and ${E}_{a}^{3:1}=16.3\pm 0.4$ and *c*_on_ = *c*_*a*_(*T*_on_).

An important property of excitations predicted by dynamic facilitation is that their duration and spatial extent remain unchanged during cooling. In Fig. 2d, we see that the mean duration of excitations Δ*t* does drop somewhat upon cooling, although this is less than one order of magnitude. Turning to the distribution of durations of excitations *P*(Δ*t*) in Fig. 2c, this presents a large exponential tail which gradually converges for temperatures well below *T*_mct_(≈0.55 for KA 2:1). We speculate that this change is related to the different mechanisms of relaxation at a higher temperature, approaching the mode-coupling crossover and at temperatures above it^13^. A similar conclusion may be reached from Fig. 2e, where we see that the distribution of displacements in excitations is unchanged with respect to temperature.

**Fig. 3:**
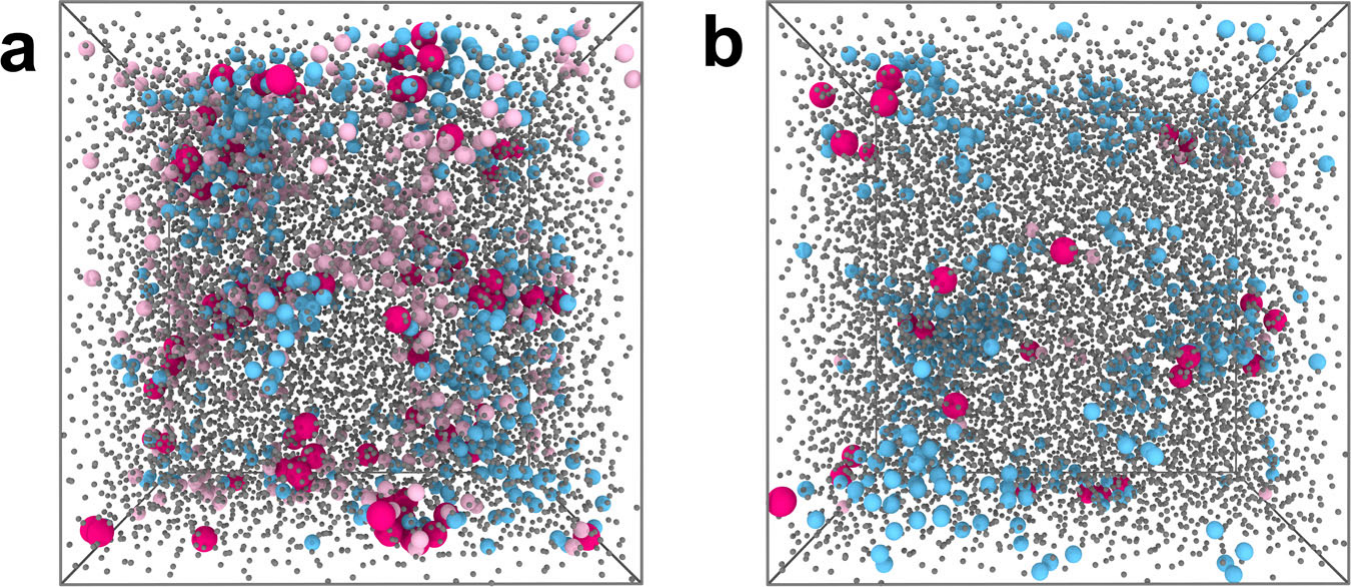
Snapshots of the KA 2:1 system. Snapshots at a temperature *T* = 0.55 ≈ *T*_mct_ (**a**) and *T* = 0.48 (**b**). Particles are identified as either in a CRR or in an excitation during a period *t*_traj_. Bright pink: particles in both CRR and excitations. Blue: CRR. Pale pink: excitations. Grey: neither. Particles are not rendered to scale.

At low temperatures, the population of particles actively moving in an excitation is less than one in a typical snapshot. Therefore in Fig. 3, we render those particles which were in an excitation during a trajectory of duration *t*_traj_. Particles in excitations during this period *t*_traj_ at the lowest temperature sampled (*T* = 0.48) are shown in pink in Fig. 3b. In Fig. 3a, we show data at *T* = 0.55(≈*T*_mct_). At this higher temperature, there are many more particles in excitations.

Dynamic facilitation theory posits that relaxation is hierarchical and that the energy barrier *E*_*a*_ for relaxation over a lengthscale *a* scales logarithmically with *a*. This can be tested by extracting *E*_*a*_ from a linear fit of $\log {c}_{a}$ with respect to 1/*T* − 1/*T*_on_ (SI Fig. S5). The ${E}_{a} \sim \log a$ relationship has been tested previously in ref. ^20^ for simulated binary mixtures equilibrated at temperatures mostly above the MCT crossover. Here, we examine the scaling by focusing on the deeply supercooled regime and plot *E*_*a*_(*a*) in Fig. 2f. We find non-monotonic behaviour in *E*_*a*_(*a*) around the particle size of the smaller particles *a* ≈ *σ*_B_. The smaller particles are more mobile and thus make up 50% to 80% of excitations depending on the temperature. We expect the non-monotonic behaviour to be related to packing effects that are not explicitly included in dynamic facilitation. These furthermore appear to be system–specific. For lengthscales larger than *σ*_B_, we find behaviour consistent with the predicted logarithmic scaling.

### Coupling of thermodynamic quantities and relaxation dynamics

**Fig. 4:**
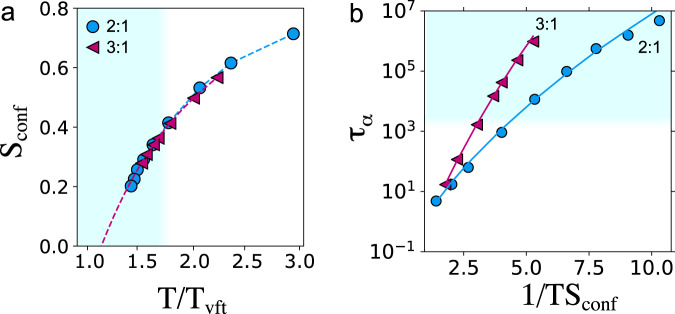
Entropy scaling in the Adam–Gibbs model and RFOT. Blue shading indicates state points past the mode-coupling crossover. **a** Configurational entropy *S*_conf_. Dashed lines are extrapolations from a fit to a quadratic function. Details of the configurational entropy are provided in section IIF of the supplementary information. **b** Testing the RFOT scaling for relaxation time *τ*_*α*_ as a function of configurational entropy *S*_conf_. Data points are fitted with the RFOT prediction Eq. (4) with the exponent *ν* as the best fit.

We now consider the thermodynamic point of view. We do so by measuring the relationship between configurational entropy and relaxation, as emphasised in the Adam–Gibbs relation and RFOT. In Fig. 4a, we find that the configurational entropy is extrapolated to vanish at a non-zero temperature *T*_k_ ≈ *T*_vft_, compatible with previous work^16^. We emphasise that such extrapolation is in no sense conclusive, and that other scenarios are possible, such as an avoided transition^33,34^.

In the Adam–Gibbs theory, the activation energy is assumed to be proportional to the volume (size) of CRR regions. The size of these regions can, in turn, be related to the inverse of the configurational entropy (a measure of the number of distinct local potential-energy minima) defined as *S*_conf_ = *S* − *S*_vib_, where *S*_vib_ is the vibrational entropy of the liquid. The relaxation time is then given by ${\tau }_{\alpha }={\tau }_{0}\exp ({A}_{{{{{{{{\rm{ag}}}}}}}}}/T{S}_{{{{{{{{\rm{conf}}}}}}}}})$ where *A*_ag_ is a constant. Supplementary Fig. 6a shows that the Adam–Gibbs relation holds well on approaching *T*_mct_ but gradually starts to break down at deeper supercooling in agreement with other studies^35^ (see SI section IID for details on the calculations). The breakdown of Adam–Gibbs theory has been related to the breakdown of the Stokes–Einstein relation for the viscosity and diffusion coefficient^36–38^. Here we confirm that for both the relaxation time and the diffusion coefficient (see SI Fig. S6b), the Adam–Gibbs relation does not hold.

A more generalised scaling compatible with RFOT has been shown to work well over a large temperature range^35^. Figure 4b shows the RFOT prediction of a modified Adam–Gibbs relation

4 $${\tau }_{\alpha }={\tau }_{0}\exp \left(\frac{{A}_{{{{{{{{\rm{rfot}}}}}}}}}}{T{S}_{{{{{{{{\rm{conf}}}}}}}}}^{\nu }}\right).$$

Treating the exponent *ν* as a fitting parameter, we find good agreement with Eq. (4) with *ν* = 0.6 ± 0.1 for KA 2:1 and 3:1.

### Cooperatively rearranging regions

**Fig. 5:**
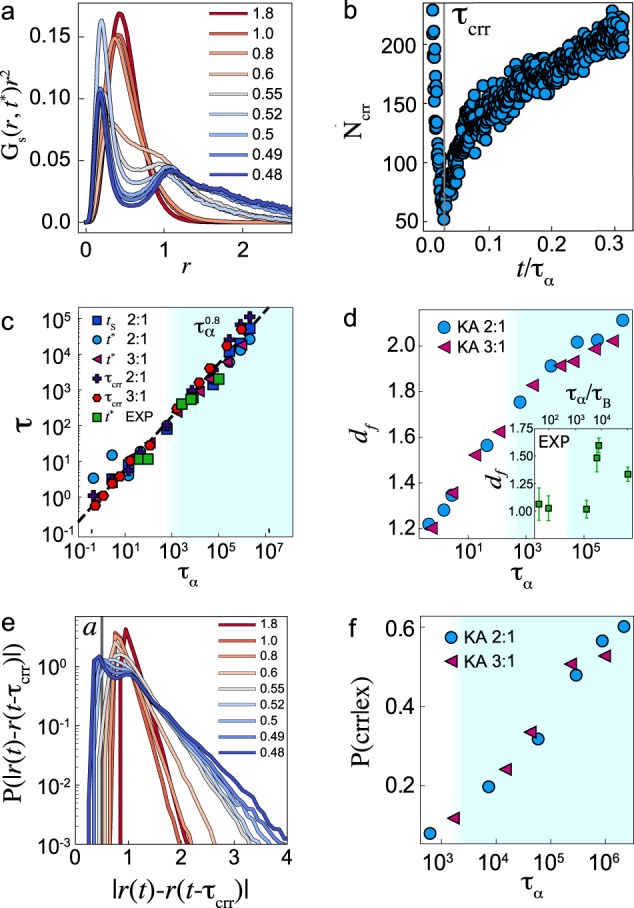
Characteristics of CRRs. Blue shading indicates state points past the mode-coupling crossover. **a** Van Hove functions for the KA 2:1 system plotted at the temperatures shown. **b** Number of clusters as a function of the chosen timescale to illustrate how *τ*_crr_ is determined. *τ*_crr_ is indicated with the grey line. **c** Different timescales as a function of the relaxation time. The times of the maximum number of particles in CRRs, *τ*_crr_ and strings *t*_s_ are shown. Also shown is t^*^, the timescale of the maximum of the non-Gaussian parameter for the KA 2:1 and 3:1 systems, as indicated. For the experiments, t^*^ is scaled with a time *t*_*f*_ for data collapse. The dashed black line is a plot of $\tau \sim {\tau }_{\alpha }^{0.8}$. **d** Fractal dimension of CRRs. Inset experimental measurements of the CRR fractal dimension. Distributions of mobilities of particles in CRRs at the temperatures indicated. **e** Distributions of mobilities of particles in CRRs at the temperatures indicated. The lengthscale *a* used to define excitations is indicated with the grey line. **f** Change of the probability that a particle that is in excitation is also part of a CRR or string with *τ*_*α*_.

Central to the Adam–Gibbs and RFOT approaches is the concept of cooperatively rearranging regions (CRRs). To identify particles which participate in CRRs, we follow ref. ^25^ and consider the self van Hove function *G*_*s*_(**r**, t*) where t* is the maximum of the non-Gaussian parameter *α*_2_ (SI Fig. S7)^25^. As seen in Fig. 5a, the van Hove function features a long tail and a crossover to two distinct “fast” and “slow” populations at deep supercooling, with the former corresponding to “hopping” events. Note that these occur on the t* timescale, while previous work at weaker supercooling also identified hopping events on much longer timescales relative to t*^39^. For the CRRs, we follow previous work^25^ and consider the subpopulation of the 5% most mobile particles. CRRs are then identified via connected clusters within such a subpopulation of fast particles, with connectivity defined by the nearest neighbour cutoff of 1.3*σ*. We find that, depending on the timescale over which we consider the displacement, the 5% most mobile particles are organised in a different manner. In particular, in Fig. 5b, we show the number of clusters as a function of waiting time for temperature *T* = 0.48. We see that there is a time at which a clear minimum in the number of clusters identified, and this allows us to also identify a timescale *τ*_crr_, the time at which the fastest 5% of the particles form the minimum number of clusters. This is numerically close to ${t}^{*} \sim {\tau }_{\alpha }^{0.8}$ [see Fig. 5c], which has been shown to be linked to the diffusion time^25^ and, therefore to decouple from *τ*_*α*_ at low temperatures.

The RFOT scenario also predicts that CRRs become progressively more compact as the temperature is decreased^1^ with a crossover around the MCT temperature. We quantify these changes through the fractal dimension *d*_*f*_ of the CRR clusters, obtained from the scaling of the number of particles with the radius of gyration (SI Fig. S8). Figure 5c shows that in simulations *d*_*f*_ increases with *τ*_*α*_ and two regimes (below and above the MCT crossover) can be detected. The experiments [Fig. 5c inset] also displays a trend toward more compact clusters. A static lengthscale has previously been obtained from our experimental data, which was shown to exhibit the scaling expected by RFOT^9^. In this work, we have not extracted a static lengthscale, which might be compatible with the point-to-set length in RFOT. However, previously such static lengthscales have been related to the configurational entropy *S*_conf_^16^. Since our measurements of *S*_conf_ in Fig. 4a show the same scaling compatible with that obtained previously, we expect that a similar behaviour for the static lengthscale would hold here.

### String analysis

**Fig. 6:**
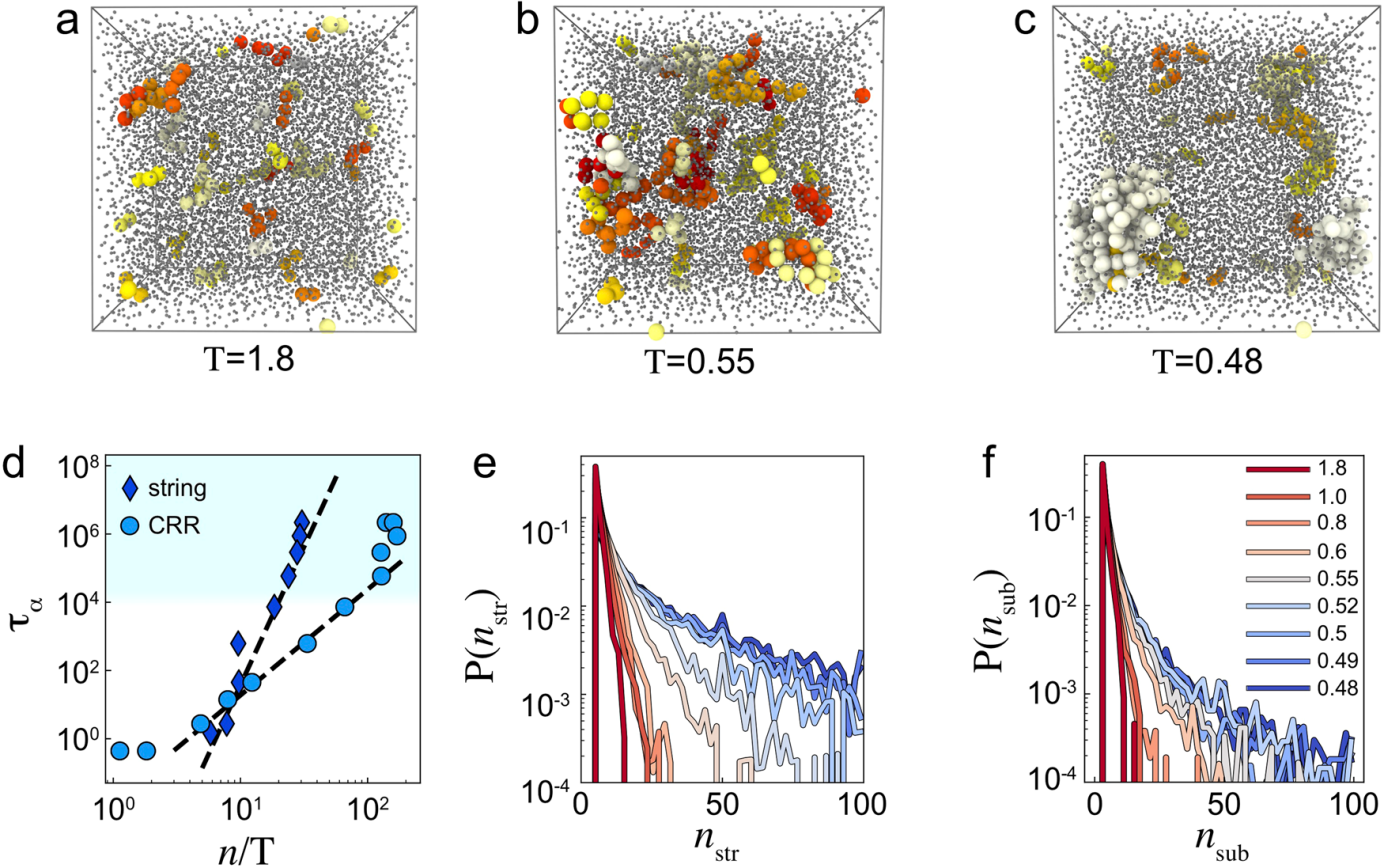
Coupling between excitations and CCRs: strings and correlations. Blue shading indicates state points past the mode-coupling crossover. **a**–**c** String analysis. Particles in distinct strings at the temperatures shown are rendered in different colours, small grey particles are not identified in strings. **d** Relaxation time *τ*_*α*_ as a function of *n* for strings and the size of CRR clusters and strings. Dashed lines are fits to Adam–Gibbs scaling for the size of clusters and strings following ref. ^40^. **e** Size distribution of full strings at different temperatures. **f** Size distribution of substrings at different temperatures. The temperatures shown in the legend in (**f**) also apply to (**e**).

Previous studies of dynamic heterogeneity found string-like propagation of mobility, which has been associated with CRRs^40^ and dynamic facilitation theory^25^. Following ref. ^41^, we first identify mobile particles that replace their neighbour on the timescale t*. The pair is considered for a string if $\min \left[|{\underline{r}}_{i}({t}{*})-{\underline{r}}_{j}(0)|,|{\underline{r}}_{j}(0){\underline{r}}_{i}({t}{*})|\right] < \delta=0.6$. Strings are constructed by connecting pairs that have a common particle involved and that occur in the same frame. As shown in Fig. 6a–c, they become more and more connected and grow with decreasing temperatures. At the lowest temperatures that we access, the strings become more significantly compact, as shown in Fig. 6a–c. The Adam–Gibbs theory predicts a linear scaling in $\log ({\tau }_{\alpha })$ and CRR size over temperature *n*/*T*. One may perform the same analysis on strings and CRRs, see Fig. 6d. Previously, a better agreement was found for the linear scaling for strings rather than CRR clusters^40^. At the deeper supercooling, we access here, we also find a good agreement for the strings, but the CRRs percolate below *T*_mct_, which complicates the comparison.

In Fig. 6e, we show the distribution of string sizes at various temperatures. We see that the tails of the distribution of the number of particles per string become larger with deeper supercooling, indicating that collective relaxation becomes more important at deep supercooling. We also calculate the time *t*_*S*_ at which the maximum of mobile particles form non-trivial strings (comprising more than two particles), as shown in Fig. 5c. This exhibits a similar scaling to t* and *τ*_crr_. To analyse the dynamics of strings in more detail, we investigate substrings. To identify these substrings, we consider the largest subset of particles in each string that move in the same frame measured on a timescale of the framerate t*/100. These substrings are smaller than the full strings, as shown by the size distribution in Fig. 6f. Thus, they represent relaxation phenomena intermediate in length– and time–scale between excitations and CRRs. By varying timescales further, along with other criteria^25^, it is possible to carry out a characterisation of relaxation events across time– and length–scales intermediate between excitations and CRRs. This is compatible with relaxation events over a range of timescales observed in very recent computer simulation results^42^.

### Locally favoured structures

**Fig. 7:**
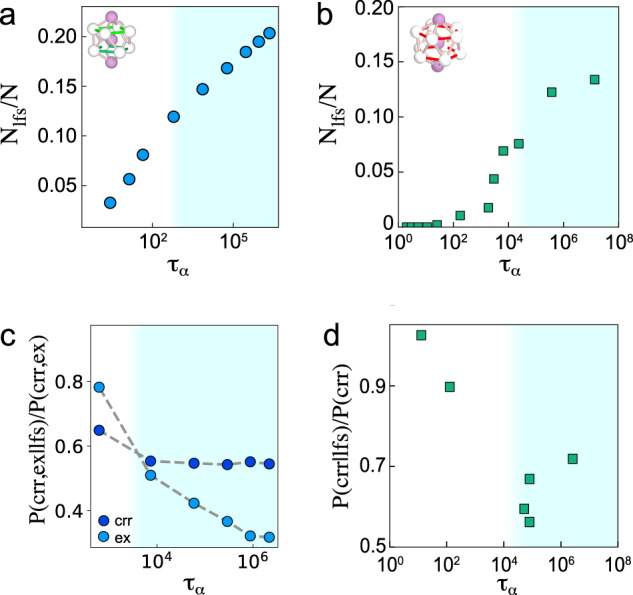
Analysis of locally favoured structures for KA 2:1 and experiments. Blue shading indicates state points past the mode-coupling crossover. The locally favoured structure (LFS) for the KA model is the bicapped square antiprism as indicated in (**a**) and, for the experiments, the icosahedron (**b**). In addition to the 4:1, this holds for the 2:1 and 3:1 compositions as well^43^. **a** Population of particles in antiprisms *N*_lfs_/*N* as a function of relaxation time for KA 2:1. **b** Population of icosahedra in experiments. **c** The probability that a particle in a CRR or excitation is also in an LFS, normalised by the probability of being in a CRR or excitation for KA 2:1. **d** The probability that a particle in a CRR is also in an LFS, normalised by the probability of being in a CRR for the experiments.

Among the key measures of structure in glassforming systems are the locally favoured structures (LFS) which have been related to the Geometric Frustration theory^8^. For the Kob–Anderson model, including the 2:1 and 3:1 compositions, the LFS is the bicapped square antiprism^43^, which is depicted in Fig. 7a, and for the experiments, the icosahedron is a suitable LFS (b)^9^. Figure 7a, b also shows the population of particles in LFS *N*_lfs_ as a function of relaxation time. We find that the population increases significantly with relaxation time, which is consistent with a number of other glassforming systems^44^.

To probe the relationship between LFS and CRRs and excitations, we consider the probability that a particle which is in an LFS is also in a CRR or excitation *P*(CRR, ex∣lfs), normalised by *P*(CRR, ex). We plot this quantity in Fig. 7c for the KA 2:1 and the relation with CRRs for the experiments (d). Since CRRs and excitations correspond to mobile particles, while LFS are expected to relate to more static particles, we expect that *P*(CRR, ex∣lfs)/*P*(CRR, ex) < 1, which we indeed find for supercooled states. We find that for excitations, there is a strong decrease upon supercooling, showing that at low temperatures, particles in LFS are very unlikely to be found in excitations. For the KA 2:1, the correlation is rather weaker for CRRs, with the experiments showing a somewhat stronger correlation. This weak correlation with respect to the excitations is likely related to the fact that over the timescale of a CRR, t*, particles can move in and out of an LFS, as investigated previously^45^. However, on the shorter timescale of excitations, it is rather more likely that particles in excitations are not in LFS.

### CRRs and excitations

Finally, we consider the CRRs in relation to the excitations. Plotting the distribution of CRR displacements *P*(∣*r*(*t*) − *r*(*t* − *τ*_crr_)∣) in Fig. 5e, we note that at the characteristic time *τ*_crr_, most of the particles in CRRs have performed displacements larger than the excitation lengthscale *a* at higher temperatures. In Fig. 5f, we compute the average fraction of excitations that are identified in a CRR at the same time and we find that this increases with the relaxation time, from below 10% to 60% at the lowest temperatures. This indicates that, as the temperature is decreased, the CRRs are increasingly more predictive for the location of excitations. Particles found in strings also follow a similar behaviour. However, we have to be careful with this interpretation since the identification of both excitations and CRRs depends on the parameters chosen. We, therefore, probe the temperature dependence upon these parameters of the particles identified in excitations and CRRs in SFig. S9. We see that choosing the 5% fastest particles over the timescale t* leads to a threshold of lower displacement as the temperature is dropped. Meanwhile, we have fixed the threshold *a* to identify particles in excitations. This observation alone is sufficient to lead to an increase in excitation particles identified in CRRs. In the future, it would be interesting to investigate alternative definitions for particles participating in CRRs, which do not fix the population.

To determine to what extent the CRRs are a consequence of the cumulative contributions of multiple excitations would require an unmanageable amount of computation, given the separation of timescales involved. However, we can perform the following estimate: if in an interval of duration *t*_traj_ we have *Nc*_*a*_ excitations, during one relaxation time, we expect to detect *Nc*_*a*_*τ*_*α*_/*t*_traj_ excitations. With the chosen *a*, this extrapolation results in more than 5% for all temperatures and more than the system size for the coldest temperatures. This would suggest that, on average, each particle participating in a CRR would have participated in many excitations on the timescale of the CRR, *τ*_crr_.

Addressing the separation between the microscopic excitation timescales and that of the CRRs thus emerges as a key challenge to understand the link between these two mechanisms and test the hypothesis of hierarchical relaxation in facilitation theory. Some work has been carried out in this direction by refs. ^42,46^. Here connected clusters of mobile particles at short timescales were shown to be related to the excess wing of *β* relaxation. How the excitations relate to defects and soft spots in such a glass^42,47^ is an intriguing topic for future investigation.

## Discussion

We have analysed particle-resolved data on the static and dynamic properties of simulated and experimental liquids at supercooling three orders of magnitude deeper than the mode-coupling crossover. We have shown that, tested against their own metrics, both dynamic facilitation and the RFOT pictures are compatible with the data. Contrary to previous reports^18^, at the deep supercooling considered here, we do not find a breakdown of the facilitated dynamics. Rather, facilitation becomes more significant as a relaxation mechanism at deeper supercooling compatible with very recent work which emphasises facilitated coupling between relaxation events at deep supercooling^42^. At temperatures below *T*_mct_ a second maximum appears in the van Hove function and the mobility. This is a sign of hopping and, to the best of our knowledge, has not previously been seen at the timescale t*, suggesting that such hopping events become more significant at deeper supercooling.

The population of excitations follows an Arrhenius–like scaling and their nature appears to undergo little change. This is compatible with the parabolic law, which accurately describes the dynamics of many glassformers^29^. Interestingly, ref. ^48^ find increased coupling between spatially separated excitations with supercooling, which may be related to the different algorithms employed in that work, which in particular used inherent states to identify excitations, unlike the thermalised configurations here. We find that the excitation energy barriers show a minimum at the particle diameter, but for higher *a* are compatible with the logarithmic scaling. The pronounced minimum is likely due to packing effects. Furthermore, we find that at deep supercooling, particles in bicapped square antiprism locally favoured structures are unlikely to be in excitations. This further corroborates the importance of packing effects for dynamic facilitation. In the future, it would be interesting to explore these kinds of predictions in an off–lattice model free from packing effects^49^.

At the same time, we observe substantial evidence for a rapid drop of the configurational entropy, too rapid for the original Adam–Gibbs relation to hold but compatible with RFOT-like models. We identified multiple instances of a crossover around the MCT temperature: in the dynamic susceptibility, in the mobility distribution, and in the fractal dimension of the cooperative regions. Our experiments confirm this picture regarding relaxation times and the fractal dimension of the CRRs. The CRRs increase their connectivity until a maximum is reached at a characteristic time that is shorter than the relaxation time at low temperatures. In our experiment and simulations, particles in LFS are somewhat less likely to be found in a CRR at deep supercooling.

We have performed an analysis which has been used previously to identify CRRs through cooperative motion^25^. At the weaker supercooling considered before for *T* ≳ *T*_mct_, this revealed string–like structures. As noted above, RFOT predicts more compact CRRs at deeper supercooling, and this we indeed find at the lowest temperatures we consider. Interestingly, the timescale for this string analysis may be varied and can reveal relaxation phenomena intermediate in length– and time–scales between microscopic excitations and larger, longer-lived CRRs. Therefore, in the future, it is possible that this string analysis could be used to bridge the length– and time–scales between excitations and CRRs.

Previous work on atomistic systems has suggested a connection between the dynamical phase transitions predicted by dynamic facilitation and the features of the thermodynamic approach linking the two through a structural–dynamical lower critical point which lies close to *T*_*k*_^50,51^. Here we have quantified the distinctive observables of the dynamic and thermodynamic approaches (excitations and cooperative regions) and showed that a connection between the two exists and becomes stronger at deep supercooling. In particular, we have found that excitations can be increasingly found in cooperative regions. Our study supports a low-temperature scenario where the short-time dynamics is well described by the facilitation mechanism. How this relates to a hierarchical relaxation, the observed decrease of configurational entropy, and “soft spots” is fertile ground for future investigation.

## Methods

### Simulation and model details

We study the binary Lennard-Jones Kob-Andersen (KA) mixture with 2:1 and 3:1 compositions in the NVT ensemble at a number density *ρ* = 1.400 using the Roskilde University Molecular Dynamics (RUMD) package^22^. The interactions of the KA mixture are defined by ${u}_{ij}(r)={\epsilon }_{\alpha \beta }[{(\frac{{\sigma }_{\alpha \beta }}{r})}^{12}-{(\frac{{\sigma }_{\alpha \beta }}{r})}^{6}]$ with parameters *σ*_*AB*_ = 0.80, *σ*_*BB*_ = 0.88 and *ϵ*_*AB*_ = 1.50, *ϵ*_*BB*_ = 0.50 (*α*, *β* = A, B). We employ a unit system in which *σ*_*AA*_ = 1, *ϵ*_*AA*_ = 1, and *m*_*A*_ = *m*_*B*_ = 1 (we refer to this as LJ units). We study system sizes *N* = 10,002 and 100,002 for KA 2:1 and *N* = 10,000 for KA 3:1. The temperature is in the range *T* = [0.48, 2.00] for KA 2:1 and *T* =  [0.68, 2.00] for KA 3:1. For the lowest temperature it took well over 1 year to equilibrate on an Nvidia 1080 GTX card. Further details of our simulations may be found in the SI.

### Experimental

Poly methylmethacrylate (PMMA) colloids with a polyhydroxystearic acid stabiliser were synthesised^52^. To enhance spatial resolution, a non-fluorescent PMMA shell was grown on the fluorescent cores to yield a total radius of 270 nm (polydispersity ≈ 8%, Brownian time *τ*_*B*_ = 34 ms). The structural relaxation time was monitored for waiting times of up to 100 days until it reached a steady state, at which point the sample was considered to be equilibrated. Samples were imaged using a Leica SP8 stimulated emission depletion microscope in STED-3D mode. As before, we determine the compressibility factor *Z* from the volume fraction with the Carnahan-Starling relation^9^. Further details of our experiments may be found in the SI.

## Supplementary information

Supplementary information

## Data Availability

Representative data is available here: 10.5281/zenodo.5704503. Further data that support the findings of this study are available from the corresponding author upon request.

## Code availability

The code developed here to detect excitations is available here: https://github.com/levkeO/detectInstantons.
