# Supplementary material for: Probing excitations and cooperatively rearranging regions in deeply supercooled liquids
Source: Nat Commun. 2023 May 5;14:2621. doi: 10.1038/s41467-023-37793-2 (PMC10163050; doi:10.1038/s41467-023-37793-2)
Supplement: Supplementary file 1 — Supplementary information [file 41467_2023_37793_MOESM1_ESM.pdf]

Supplementary information: Probing excitations and cooperatively rearranging regions in deeply supercooled liquids

This supplementary information is organised into three main sections. Section I details our simulation and experimental methodologies. Analysis methods are described in section ??. In addition to frequently determined quantities like the structural relaxation time, dynamic susceptibility, non–Gaussian parameter, and mode–coupling temperature, in section I E we describe the algorithm that we use to identify excitations. The configurational entropy is determined as described in section I F. This is accompanied by additional figures and finally, we present the parameters from all the fits in this work in tables Supplementary Fig.1 and Supplementary Fig.2.

I. SUPPLEMENTARY METHODS

A. Simulation and model details

Here we use the Roskilde University Molecular Dynamics (RUMD) code. This simulates standard Molecular Dynamics, taking advantage of the many core nature of graphics cards to achieve high performance. In this way, the dynamics simulated are realistic, which is useful for estimating the dynamical quantities of interest here [1, 2]. While the dynamics are realistic, the configurations generated are limited to around three decades of increase in relaxation time with respect to the mode–coupling crossover. Unlike, e.g., SWAP which is an unphysical algorithmic improvement to simulate low temperatures, RUMD achieves high performance by optimising the force calculation to utilise thousands of cores available on the GPU. RUMD thus simulates the "true" dynamics of the system as opposed to, e.g., the SWAP algorithm.

We study the binary Lennard-Jones Kob-Andersen (KA) mixture at the non-standard 2:1 and 3:1 composition in the NVT ensemble (Nose-Hoover thermostat) at $\rho = 1.400$ using the Roskilde University Molecular Dynamics (RUMD) package [3]. We also simulate KA 4:1 for comparing our algorithm's with data in the literature. The interatomic interactions of the KA mixture are defined by

$$v_{ij}(r) = \epsilon_{\alpha\beta}\big[(\frac{\sigma_{\alpha\beta}}{r})^{12} - (\frac{\sigma_{\alpha\beta}}{r})^{6}\big] \tag{1}$$

with parameters $\sigma_{AB} = 0.80$, $\sigma_{BB} = 0.88$ and $\epsilon_{AB} = 1.50$, $\epsilon_{BB} = 0.50$ (α, β = A, B). The pair potential is cut and shifted at $r_c = 2.5\sigma_{\alpha\beta}$. We employ a unit system in which $\sigma_{AA} = 1$, $\epsilon_{AA} = 1$, and $m_A = m_B = 1$ (we refer to this as MD or LJ units). We study system sizes $N = 10{,}002$ and 100,002 for KA 2:1 and $N = 10{,}000$ and 100,000 for KA 3:1. The time step is $\Delta t = 0.0025$ or 0.0035 depending on the temperature. The temperature is in the range $T =$ [0.48, 2.00] for KA 2:1 and $T =$ [0.68, 2.00] for KA 3:1, and at the lowest temperature required well over 1 year to equilibrate on a Nvidia 1080 GTX card. We confirmed equilibrium by running the simulations back-to-back at least twice and comparing the intermediate scattering functions.

More specifically, the systems were quenched from a high temperature configuration using a thermostat and equilibrated at low temperatures according to the previously mentioned criteria. As the low temperature simulations require

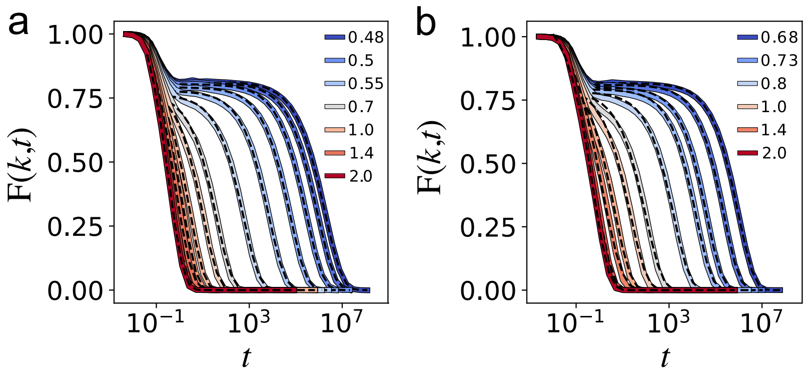

Supplementary Figure 1. Intermediate scattering functions plotted for a wavevector close to the main peak of the static structure factor. These are fitted with a stretched exponential for (a) KA 2:1 and (b) KA 3:1. Coloured lines are data at the temperatures indicated in the legend, dashed black lines are fits.

absolute stability against round errors occurring over many months it is not recommended to use NVE simulations for the equilibration nor production runs but rather NVT simulations which eliminate any entropic drift in energy due to round off errors.

B. Experimental

Fluorescent dyed-poly methylmethacrylate (PMMA) colloids with a polyhydroxystearic acid comb stabiliser were synthesised using established methods [4]. To enhance spatial resolution between particles, a non-fluorescent PMMA shell was grown on the fluorescent cores and cross-linked with ethylene glycol dimethacrylate (EGDM) to yield a total radius of 270 nm (polydispersity $\approx 8\%$, Brownian time $\tau_B = 34$ ms). PMMA particles were imaged in a density– and refractive index matching mixture of cis-decalin and cyclohexyl bromide.

Samples were loaded into cells constructed of three coverslips on a microscope slide, where two of the coverslips acted as a spacer, and sealed using epoxy. The structural relaxation time was monitored for waiting times of up to 100 days until it reached a steady state at which point the sample was considered to be equilibrated [5]. For example, the state point $\phi = 0.598$ reached equilibrium after 16 days, corresponding to more than $10\tau_\alpha$. Samples were imaged using a Leica SP8 inverted stimulated emission depletion microscope with a 100x oil immersion lens in STED-3D mode, mounted on an optical table. Measurements were taken at least 20 particle diameters from the cell wall to minimise any structural or dynamic influence of surface layering, which typically persisted for around 5 particle diameters. Images were deconvolved with Huygens Professional version 15.05 prior to analysis (Scientific Volume Imaging, The Netherlands, `http://svi.nl`).

C. Structural Relaxation Time and Dynamic susceptibility

The relaxation time τ_α is calculated from the self-part of intermediate scattering function $F_S(k,t)$ for a wave vector near the first peak of the A-particle static structure factor. $F_S(k,t)$ is fitted with a stretched exponential:

$$F_S(k,t) = A \exp\left[\left(-\frac{t}{\tau_\alpha}\right)^\beta\right] \tag{2}$$

where A is a constant and $\beta \leq 1$ is the stretching exponent. Intermediate scattering functions for KA 2:1 and 3:1 are shown in Fig. 1.

$$\chi_4(t) \equiv \frac{1}{N}\big(\langle q(t)^2\rangle - \langle q(t)\rangle^2\big) \tag{3}$$

The dynamic susceptibility is calculated from the self-part of the overlap function where

$$q(t) = \sum_{i=1}^{N} \Theta\big(r_a - |\mathbf{r}_i(t+t_0) - \mathbf{r}_i(t_0)|\big) \tag{4}$$

Here Θ is the Heaviside step function and $r_a = 0.30$. The same value is used for both species of particles. The dynamic susceptibility for KA 2:1 is shown in Fig. 1(c) in the main text.

D. Determining the Mode–Coupling Temperature

The mode-coupling temperature is around $T_{\rm mct} = 0.55$ for KA 2:1 and 0.7 for KA 3:1 (see Fig. 2). The mode coupling temperature $T_{\rm mct}$ is estimated with a power law fit [6].

$$\tau_\alpha = B\left(\frac{1}{T_{\rm mct}} - \frac{1}{T}\right)^{-\gamma} \tag{5}$$

where B is a constant.

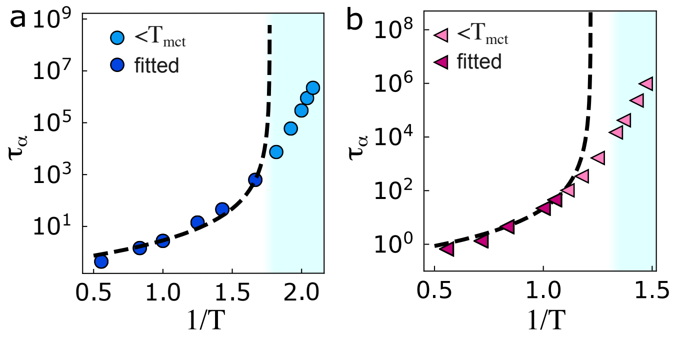

Supplementary Figure 2. Power law fit of the relaxation time to estimate T_{mct} for KA 2:1 (a) and 3:1 (b). Blue shading indicates deeper supercooling than the MCT crossover. The plot is fitted with Eq.Supplementary Fig.5 (dashed black line). Since the data is expected to go beyond T_{mct} some data was excluded from the fit as indicated.

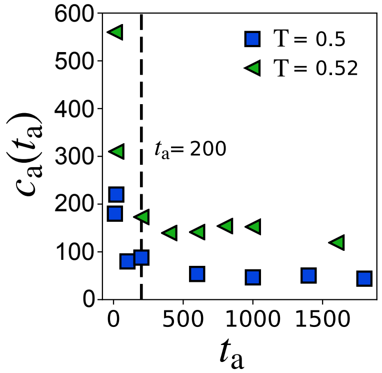

Supplementary Figure 3. Detected excitations in the KA 2:1 system depending on the choice of t_a the lowest value $t_a = 200$ in the constant region is chosen as indicated by the black vertical dashed line.

E. Excitation Detection Algorithm

To identify the excitations the timescale t_a and the length scale a have to be chosen. a has to be small enough that particles can jump the distance but large enough to exclude small fluctuations. After setting $a = 0.5$ the algorithm is used for different t_a. Fig. 3 shows that there is a range where the concentration of excitations does not depend on t_a. The smallest t_a in this range was chosen ($t_a = 200\,\mathrm{LJ}$ units) as indicated by the black dashed line.

The algorithm is implemented as follows. Trajectories of length $t_{\mathrm{traj}} = 5t_a$ containing 1000 frames are generated and each particle is tested as a potential excitation. For this the average particle positions of the first and the last t_a of the trajectory are compared and if this is smaller than a the particle is rejected. This tests if the particle has committed to a new position (step 1).

If the trajectory has not been rejected we check for each frame if the average positions in the previous t_a and the following t_a are at least a apart. This makes sure that we exclude particles that move slowly and steadily since we do not consider these to be excitations. This is important at higher temperatures but almost irrelevant at lower temperatures (step 2). To get a first estimate for the hyperbolic tangent fit, for every identified frame f that fulfills the above condition, we go forwards and backwards in time and identify the closest frame for which the distance of the position for the current frame is larger than $a/2$ (step 3). The time between those two frames is the first guess for Δt and the central frame of the excitation that produces the shortest δt guess serves as an input for the centre of the fit (step 4). The trajectory is smoothed with a spline (step 5)and a fitted with a hyperbolic tangent function to extract the exact centre of the excitation and δt (step 6). If this fit fails, the trajectory is rejected as well.

This algorithm becomes less effective when the temperature is raised to around T_{mct} since all particles move considerably on short timescales and excitations are not clearly defined. For low temperatures well below T_{mct} this algorithm is computationally not too expensive since most particles are already rejected in the first step so that the more costly procedure (moving window, fit) are only performed on very few particles.

Excitations where the fit gives a $\Delta t > t_a$ are excluded since the fit does not make much sense in that case. This

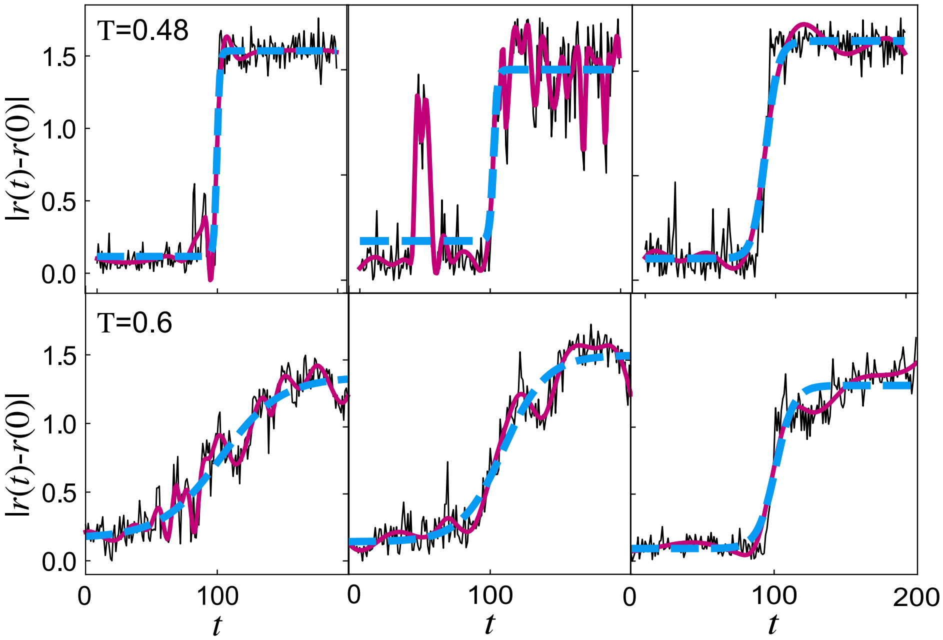

Supplementary Figure 4. Typical trajectories for the highest and the lowest temperature illustrating some examples of trajectories for low (top) and higher (bottom) temperatures for the KA 2:1 system. Black line; original trajectory. Red line: smoothed trajectory. Dashed blue line: tanh fit. See section I E for details.

algorithm only reliable detects excitations at temperatures lower than T_{mct} since above that excitations (according to our definition) are not as cleanly defined anymore since other types of movement occur (see Fig. 4). Thus we focus on the temperature range $T \lesssim T_{\mathrm{mct}}$. Since the data in Keys et al. [7] is either out of equilibrium or in the regime $T \gtrsim T_{\mathrm{mct}}$, it is difficult to compare some quantities directly.

Steps in the algorithm

1. for all particles if: $\frac{1}{t_a}\left(\sum_{t=4t_a}^{5t_a}\mathbf{r}(t) - \sum_{t=0}^{t_a}\mathbf{r}(t)\right) < a$ reject
2. else: for every frame f: if $\frac{1}{t_a}\left(\sum_{t=f-t_a}^{f}\mathbf{r}(t) - \sum_{t=f}^{f+t_a}\mathbf{r}(t)\right) > a$
3. for every frame f fullfilling these criteria:
   $\delta_{\mathrm{temp}} = \min(\mathbf{r}(t))_{\mathbf{r}(t)-\mathbf{r}(f)>a/2;t} - \max(\mathbf{r}(t))_{\mathbf{r}(f)-\mathbf{r}(t)>a/2;t<f}$
4. use frame with $\min \delta_{\mathrm{temp}}$ as initial guess for tanh fit
5. smooth trajectory with spline
6. fit trajectory with $\tilde{a}\tanh\left((t-t')/\Delta t\right)$ to get Δt or reject if fit fails

F. Configurational entropy

The configurational entropy S_{conf} is calculated from the total entropy S by subtracting the basin entropy in the harmonic approximation [8, 9]

$$S_{\mathrm{conf}} = S - S_{\mathrm{vib}} \tag{6}$$

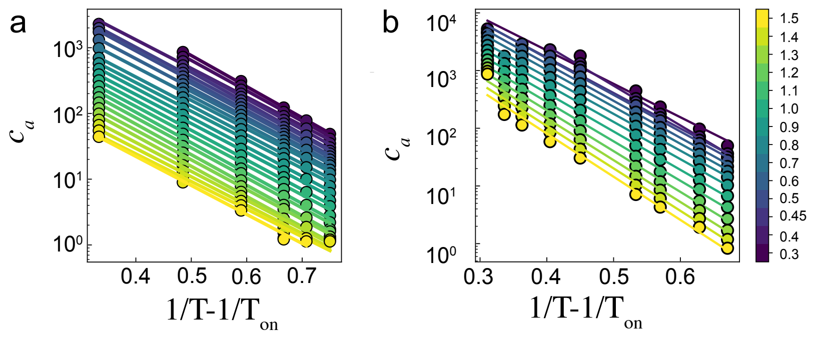

Supplementary Figure 5. Fitting the excitation population c_a as a function of inverse temperature to obtain the excitation size a dependence of the excitation energy E_a. (a) KA 2:1 for $T < T_{\mathrm{mct}}$ (b) KA 3:1 for $T < T_{\mathrm{mct}}$.

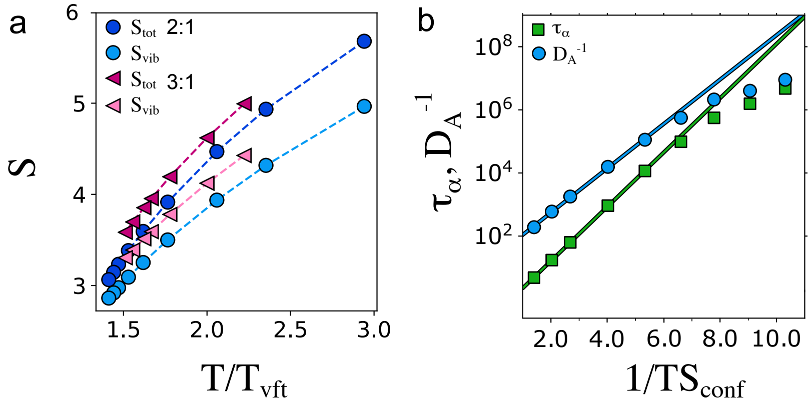

Supplementary Figure 6. Entropy scaling in the Adam-Gibbs model and RFOT. The blue shading denotes state points at lower temperatures than the mode-coupling crossover. (a) The total entropy S and vibrational entropy S_{vib} as a function of T/T_{vft}. (b) The relaxation time τ_α and diffusion coefficient D_{A}^{-1} as functions of $1/TS_{\mathrm{conf}}$ for the 2:1 model. The Adam-Gibbs relation breaks down below T_{mct}. Lines are linear fits to test the Adam-Gibbs relation as discussed in the main text.

where

$$S_{\mathrm{vib}} = \Big\langle \sum_{a=1}^{3N} (1 - \ln(\beta \hbar \omega_{\mathrm{a}}) \Big\rangle_{\mathrm{IS}} \tag{7}$$

In this equation $\beta = 1/k_{\mathrm{B}}T$ and ω_{a}^2 is an eigenvalue of the Hessian matrix for the inherent state. The average is taken over 200 inherent states with about τ_α in between the configurations. Inherent states are found by a simple gradient descent method which works well for the KA liquid.

The total entropy S is calculated via thermodynamic integration from low density (the ideal gas limit) (see Fig. 6). Details of the calculation can be found in Ref. [10]. The definition of the total entropy in MD units requires a value for Planck's constant in MD units. We used $h^* = 0.1857$ which is calculated from using $\sigma = 0.3405 \cdot 10^{-9}$ m and $\epsilon = 0.03994$ kg/mol when converting to MD units [9].

The diffusion coefficient is determined from the long-time limit of the A-particle mean-square displacement.

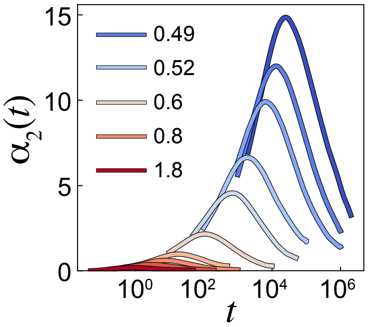

Supplementary Figure 7. Non–Gaussian parameter $\alpha_2(t)$ for different temperatures for the KA 2:1 system as defined in the legend.

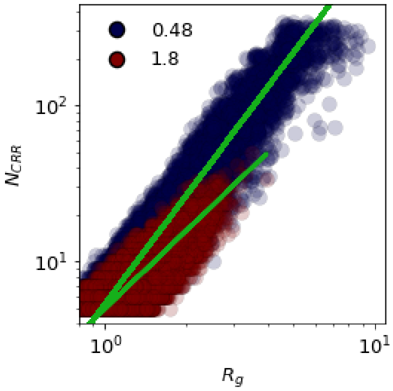

Supplementary Figure 8. Number of particles in CRRs as a function of their radius of gyration. Data are shown for temperatures of 0.48 and 1.8 are temperatures with data points in blue and red for as indicated. Points are data and the green lines are orthogonal linear fits. The fractal dimension is the exponent of power law fit.

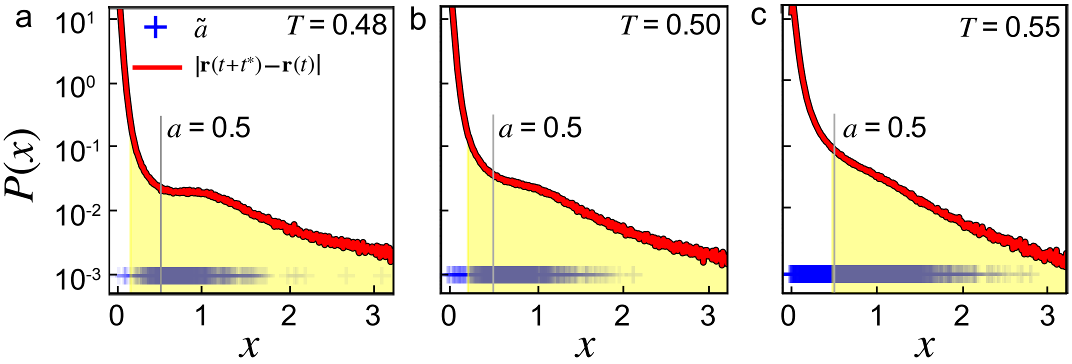

Supplementary Figure 9. Comparison of displacements in CRRs and excitations at three selected temperatures: (a) Temperature $T = 0.48$. (b) $T = 0.50$. (c) $T = 0.55$. Plotted is the distribution of displacements of particles on the maximum of the non–Gaussian parameter timescale t^* (red line). Yellow shaded regions denotes 5% largest displacements which we use to define particles in CRRs. Jump sizes of particles during an excitation event are indicated as blue crosses. Note that the some of the blue crosses lie at values less that a, the nominal jump displacement. For this there are (at least) two reasons: firstly, a is a fit parameter, the best fit does not always capture the full size of the jump. Secondly it is calculated using the displacement from frame zero, so the part of the trajectory that is fitted could move parallel to the origin so that the fit is much less than a although the displacement between the two is larger than a.

Supplementary Table I. Exponent ν from the RFOT fit Eq. 8 and T_{mct} estimated with a power law fit Eq. Supplementary Fig.5.

	KA 2:1	KA 3:1
ν	0.6 ± 0.1	0.6 ± 0.1
T_K	0.39 ± 0.2	0.51 ± 0.3
T_{mct}	0.55 ± 0.09	0.7 ± 0.1

Supplementary Table II. Parameters fitted to the various systems considered.

		KA 2:1	KA 3:1	Experiments
VFT fit	T_{vft} or Z_{vft}	0.34 ± 0.01	0.45 ± 0.01	32 ± 1
	τ_0	0.08 ± 0.02	0.02 ± 0.01	0.9 ± 0.2
	D	21 ± 1	20 ± 1	$2.8 \pm 0.6 \times 10^{-3}$
parabolic fit	T_{on} or Z_{on}	0.75 ± 0.04	1.2 ± 0.2	23 ± 3
	τ_{on}	$(5 \pm 1) \cdot 10^1$	$(3 \pm 1) \cdot 10^1$	$(1 \pm 1) \cdot 10^5$
	J	4.3 ± 0.3	5.0 ± 0.4	0.667 ± 0.9

[1] Berthier, L. & Reichman, D. Modern computational studies of the glass transition. *ArXiV, to appear in Nature Rev. Phys.* 2208.02206 (2022).

[2] Bailey, N. P. *et al.* Rumd: A general purpose molecular dynamics package optimized to utilize gpu hardware down to a few thousand particles. *arXiv* 1506.05094 (2015).

[3] Bailey, N. *et al.* RUMD: A general purpose molecular dynamics package optimized to utilize GPU hardware down to a few thousand particles. *SciPost Phys.* **3**, 038 (2017).

[4] Elsesser, M. T., Hollingsworth, A. D., Edmond, K. V. & Pine, D. J. Large Core-Shell Poly(methyl methacrylate) Colloidal Clusters: Synthesis, Characterization, and Tracking. *Langmuir* **27**, 917–927 (2011).

[5] El Masri, D. *et al.* Dynamic light scattering measurements in the activated regime of dense colloidal hard spheres. *J. Stat. Mech.* **2009**, P07015 (2009).

[6] Charbonneau, P. & Reichman, D. Mode-coupling theory. *J. Stat. Mech.: Theory and Experiment* P05013 (2005).

[7] Keys, A. S., Hedges, L. O., Garrahan, J. P., Glotzer, S. C. & Chandler, D. Excitations Are Localized and Relaxation Is Hierarchical in Glass-Forming Liquids. *Phys. Rev. X* **1**, 021013 (2011).

[8] Berthier, L., Ozawa, M. & Scalliet, C. Configurational entropy of glass-forming liquids. *J. Chem. Phys.* **150**, 160902 (2019).

[9] Sengupta, S. *Investigations of the role of spatial dimensionality and interparticle interactions in model glass-formers*. Ph.D. thesis, Theoretical Sciences Unit Jawaharlal Nehru Centre for Advanced Scientific Research (2013).

[10] Bell, I. H., Dyre, J. C. & Ingebrigtsen, T. S. Excess-entropy scaling in supercooled binary mixtures. *Nat Commun* **11**, 4300 (2020).
